# HIC1 links retinoic acid signalling to group 3 innate lymphoid cell-dependent regulation of intestinal immunity and homeostasis

**DOI:** 10.1371/journal.ppat.1006869

**Published:** 2018-02-22

**Authors:** Kyle Burrows, Frann Antignano, Alistair Chenery, Michael Bramhall, Vladimir Korinek, T. Michael Underhill, Colby Zaph

**Affiliations:** 1 The Biomedical Research Centre, University of British Columbia, Vancouver, British Columbia, Canada; 2 Department of Pathology and Laboratory Medicine, University of British Columbia, Vancouver, British Columbia, Canada; 3 Infection and Immunity Program, Monash Biomedicine Discovery Institute, Monash University, Clayton, Victoria, Australia; 4 Department of Biochemistry and Molecular Biology, School of Biomedical Sciences, Monash University, Clayton, Victoria, Australia; 5 Department of Cell and Developmental Biology, Institute of Molecular Genetics, Academy of Sciences of the Czech Republic, Prague, Czech Republic; 6 The Department of Cellular & Physiological Sciences, University of British Columbia, Vancouver, British Columbia, Canada; University of Medicine & Dentistry New Jersey, UNITED STATES

## Abstract

The intestinal immune system must be able to respond to a wide variety of infectious organisms while maintaining tolerance to non-pathogenic microbes and food antigens. The Vitamin A metabolite all-*trans*-retinoic acid (atRA) has been implicated in the regulation of this balance, partially by regulating innate lymphoid cell (ILC) responses in the intestine. However, the molecular mechanisms of atRA-dependent intestinal immunity and homeostasis remain elusive. Here we define a role for the transcriptional repressor Hypermethylated in cancer 1 (HIC1, ZBTB29) in the regulation of ILC responses in the intestine. Intestinal ILCs express HIC1 in a vitamin A-dependent manner. In the absence of HIC1, group 3 ILCs (ILC3s) that produce IL-22 are lost, resulting in increased susceptibility to infection with the bacterial pathogen *Citrobacter rodentium*. Thus, atRA-dependent expression of HIC1 in ILC3s regulates intestinal homeostasis and protective immunity.

## Author summary

Innate lymphoid cells (ILCs) are emerging as important regulators of immune responses at barrier sites such as the intestine. However, the molecular mechanisms that control this are not well described. In the intestine, the Vitamin A metabolite all-*trans*-retinoic acid (atRA) has been shown to be an important component of the homeostatic mechanisms. In this manuscript, we show that the atRA-dependent transcription factor Hypermethylated in cancer 1 (HIC1, ZBTB29) is required for ILC homeostasis and function in the steady state as well as following infection with the bacterial pathogen *Citrobacter rodentium*. Thus, HIC1 links RA signalling to intestinal immune responses. Further, our results identify HIC1 as a potential target to modulate ILC responses in vivo in health and disease.

## Introduction

The intestinal immune system is held in a tightly regulated balance between immune activation in response to potential pathogens and the maintenance of tolerance to innocuous antigens, such as food and commensal flora. Disruption of this balance can lead to the development of serious inflammatory disorders, such as food allergy or inflammatory bowel disease (IBD). A complex network of different immune cell types including dendritic cells (DCs), macrophages, innate lymphoid cells (ILCs), and T cells, are essential for both the induction of active immunity and the maintenance of intestinal homeostasis.

The vitamin A metabolite all-*trans*-retinoic acid (atRA) plays an important role in shaping intestinal immunity by regulating both the innate and adaptive immune systems. atRA that is generated by the metabolism of Vitamin A by intestinal epithelial cells (IECs) and a subset of CD103-expressing intestinal dendritic cells (CD103^+^ DCs) has been shown to directly affect the localization and function of lymphocytes. For example, atRA has been shown to induce expression of chemokine receptors (CCR9) and integrins (α4 and β7) that are associated with homing to, and retention in, the intestinal microenvironment [1–3]. In addition, atRA has been shown to control the balance of regulatory T (T_reg_) cells and CD4^+^ T helper 17 (T_H_17) cells in the intestine by promoting T_reg_ cell differentiation and inhibiting T_H_17 cell development [4–9]. Similarly, atRA controls the development of ILC subsets in the intestine, as mice raised on a Vitamin A-deficient (VAD) diet display reduced numbers of ILC3s [10,11], with one study showing a concomitant increase in ILC2 numbers and enhanced type 2 immunity within the intestine [10]. In addition, intestinal DC differentiation is influenced by atRA as mice raised on a VAD diet display reduced numbers of CD103^+^ CD11b^+^ DCs [12,13]. Thus, atRA-dependent processes are central to the function of intestinal T_H_ cells, ILCs, and DCs in vivo. However, the molecular mechanisms downstream of atRA signaling that control immune cell function and homeostasis remain unknown.

Hypermethylated in cancer 1 (HIC1, ZBTB29) is a transcriptional factor that was first identified as a gene that is epigenetically silenced in a variety of human cancers [14,15]. HIC1 has been shown to regulate cellular proliferation, survival and quiescence in multiple normal and tumour cell lines [16–19]. HIC1 is a member of the POZ and Kruppel/Zinc Finger and BTB (POK/ZBTB) family of transcription factors that consists of regulators of gene expression that are critical in a variety of biological processes [20]. Importantly, several members of the POK/ZBTB family are key regulators in immune cell differentiation and function, including: BCL6, PLZF and ThPOK [21–25]. Recently, we identified HIC1 as an atRA responsive gene in intestinal T_H_ cells and demonstrated a T cell-intrinsic role for HIC1 in the regulation of intestinal homeostasis as well as in development of several models of intestinal inflammation [26].

In this study, we show that deletion of HIC1 in hematopoietic cells results in a significant reduction in the number of αβ and γδ T cells, CD11b^+^ CD103^+^ DCs, and ILC3s in the intestine, resulting in susceptibility to infection with the bacterial pathogen *Citrobacter rodentium*. Although loss of HIC1 expression in T cells or CD11c^+^ cells had no effect on immunity to *Citrobacter*, deletion of HIC1 in RORγt-expressing ILC3s resulted in susceptibility to infection, due to a reduction in IL-22 production. These results identify a central role for atRA-dependent expression of HIC1 in ILC3s in the regulation of intestinal immune responses.

## Results

### Hematopoietic cell-specific expression of HIC1 controls intestinal immune cell homeostasis

**Fig 1:**
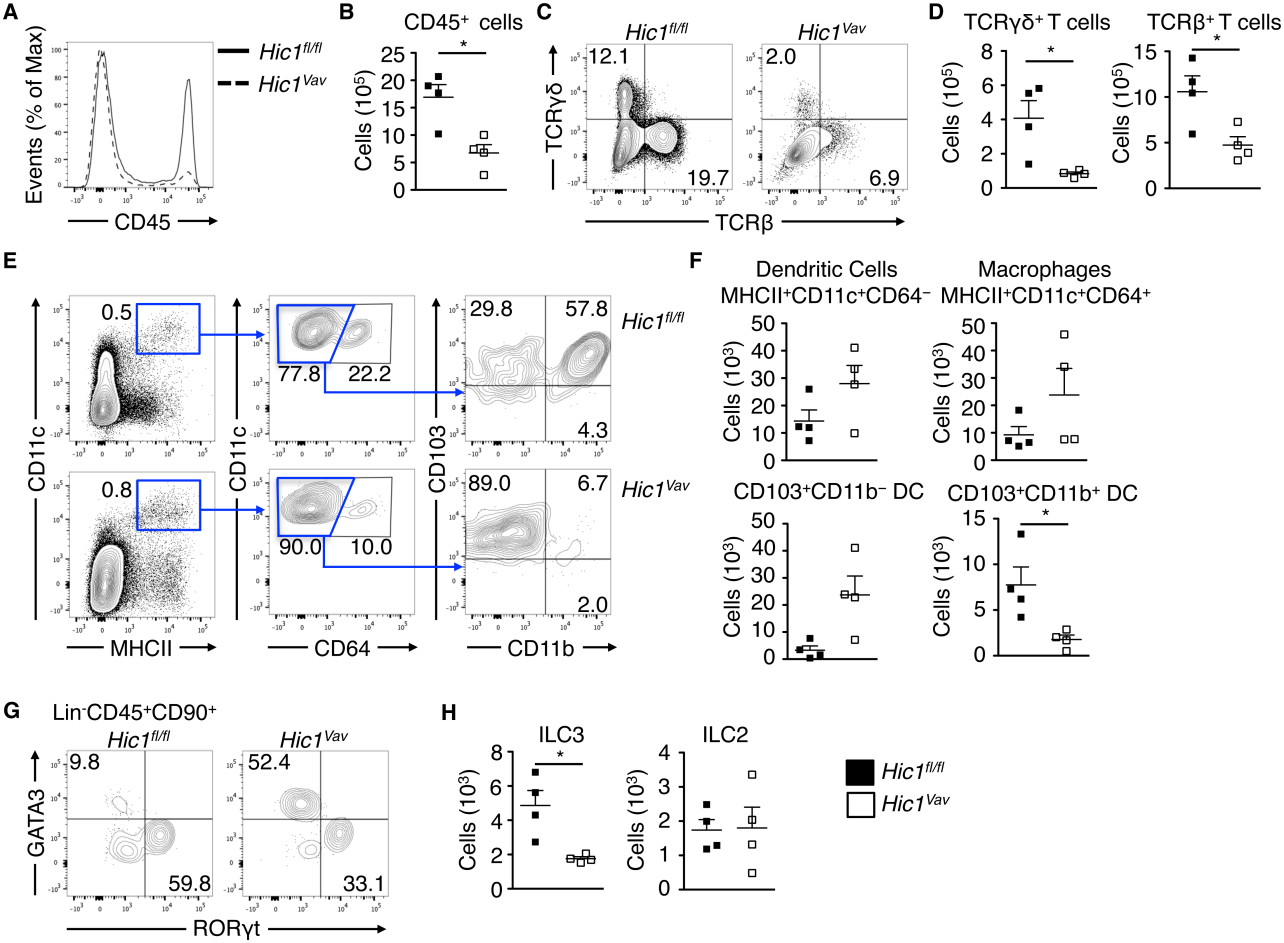
*Hic1* is required for intestinal immune homeostasis. Intestinal lamina propria (LP) cells from *Hic1*^*Vav*^ and *Hic1*^*fl/fl*^ mice at steady state were analyzed by flow cytometry to enumerate populations of: (A, B) CD45^+^ leukocytes, (C, D) TCRβ^+^ and TCRγσ^+^ T cells, (E, F) CD11c^+^ MHCII^+^ CD64^+^ macrophages, CD11c^+^ MHCII^+^ CD64^-^ DCs, (G, H) RORγt^+^ ILC3s, GATA3^+^ ILC2s. Data pooled from 2 independent experiments (***n*** = 4 per group). *, P < 0.05; Mann-Whitney test. Error bars indicate SEM.

We have previously shown that HIC1 is expressed in a wide variety of immune cells in the intestinal microenvironment, and that deletion of HIC1 specifically in T cells resulted in a severe reduction in the frequency and number of CD4^+^ and CD8^+^ T cells in the intestine [26]. To test if HIC1 played a general role in intestinal immune cell homeostasis, we generated mice with a hematopoietic cell-specific deletion of *Hic1* (*Hic1*^*Vav*^ mice) by crossing mice with *loxP* sites flanking the *Hic1* gene (*Hic1*^*fl/fl*^ mice) with mice that express the Cre recombinase under control of the *Vav* promoter (*Vav*-Cre mice). Hematopoietic cell-specific deletion of HIC1 resulted in ~50% reduction in the number of CD45^+^ cells in the intestinal lamina propria (LP) (Fig 1A and 1B). Consistent with our previous study [26], we found reduced frequencies and numbers of γδ and αβ T cells in the LP of *Hic1*^*Vav*^ mice (Fig 1C and 1D). Further, analysis of macrophage and DC populations in the LP of *Hic1*^*fl/fl*^ and *Hic1*^*Vav*^ mice revealed a specific requirement for HIC1 in CD103^+^ CD11b^+^ DCs (Fig 1E and 1F), which is consistent with previous studies identifying a role for atRA in the regulation of this DC subset [12,13]. We also found a specific reduction in the frequency and number of ILC3s in the absence of HIC1, while number of ILC2s were unaffected by the loss of HIC1 in the hematopoietic cell compartment (Fig 1G and 1H). Thus, HIC1 expression is critical for regulation of specific immune cell populations in the LP.

### Hematopoietic specific deletion of HIC1 results in susceptibility to intestinal bacterial infection

**Fig 2:**
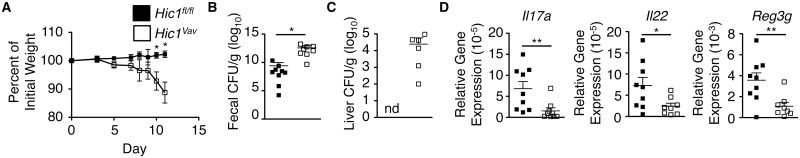
Hematopoietic deficiency of HIC1 results in susceptibility to *Citrobacter rodentium* infection. *Hic1*^*Vav*^ and *Hic1*^*fl/fl*^ mice were orally inoculated with *C*. *rodentium*. (A) Weight loss (percentage of initial weight) was calculated for each mouse over course of infection. (B, C) Bacterial loads (CFU/g) from fecal pellets (B) and liver (C) were measured at 11 days post inoculation. (D) Quantitative RT-PCR was performed to determine expression of *Il17a*, *Il22* and *Reg3g* from distal colon tissue 11 days post inoculation. Data are pooled from 2 independent experiments (*n* = 8–9 per group). *, P < 0.05; **, P < 0.01; Mann-Whitney test. Error bars indicate SEM. nd, none detected.

To directly test the role of hematopoietic cell-specific deletion of HIC1, we infected *Hic1*^*fl/fl*^ and *Hic1*^*Vav*^ mice with attaching and effacing intestinal bacterial pathogen *Citrobacter rodentium*. Following infection with *C*. *rodentium*, *Hic1*^*Vav*^ mice exhibited enhanced weight loss and significantly higher bacterial burdens in the feces compared to *Hic1*^*fl/fl*^ controls (Fig 2A and 2B). Furthermore, infected *Hic1*^*Vav*^ mice–but not *Hic1*^*fl/fl*^ mice–had dissemination of bacteria to the liver (Fig 2C), demonstrating a significant impairment in the intestinal barrier following infection. Associated with impaired bacterial containment and clearance were reduced levels of transcripts for the cytokines *Il17a* and *Il22*, as well as the intestinal antimicrobial peptide *Reg3g* (Fig 2D). Thus, expression of HIC1 within hematopoietic cells is critical to mount a proper immune response against *C*. *rodentium*.

### Loss of HIC1 in T cells or DCs does not affect immunity to *C*. *rodentium*

**Fig 3:**
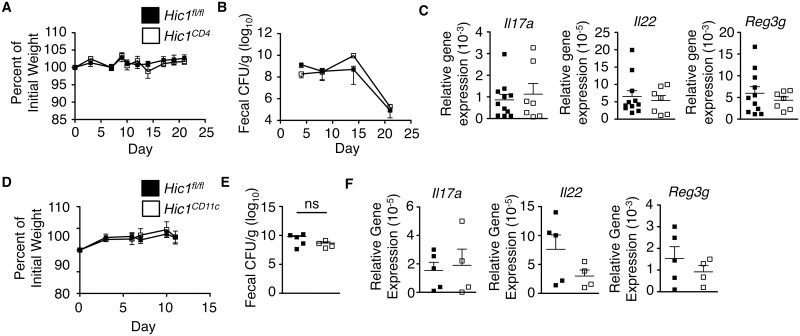
*Hic1* expression in T cells and dendritic cells is not required for immunity to *Citrobacter rodentium* infection. (A–C) *Hic1*^*CD4*^ and *Hic1*^*fl/fl*^ mice were orally inoculated with *C*. *rodentium*. (A) Weight loss (percentage of initial weight) was calculated for each mouse over course of infection. (B) Bacterial loads (CFU/g) from fecal pellets were measured over course of infection. (C) Quantitative RT-PCR was performed to determine expression of *Il17a*, *Il22* and *Reg3g* from distal colon tissue 14 days post inoculation. (D–F) *Hic1*^*CD11c*^ and *Hic1*^*fl/fl*^ mice were orally inoculated with *C*. *rodentium*. (D) Weight loss (percentage of initial weight) was calculated for each mouse over course of infection. (E) Bacterial loads (CFU/g) from fecal pellets were measured over course of infection. (F) Quantitative RT-PCR was performed to determine expression of *Il17a*, *Il22* and *Reg3g* from distal colon tissue 11 days post inoculation. (A-C) Data are pooled from 3 independent experiments (*n* = 7–11 per group). (D-F) Data are pooled from 2 independent experiments (*n* = 4–5 per group) *, P < 0.05; Mann-Whitney test. Error bars indicate SEM. ns, not significant.

As T cells, CD103^+^ CD11b^+^ DCs and ILC3s are all important in initiating and propagating ILC3/T_H_17 responses in the intestine [27–30] and these population are perturbed in *Hic1*^*Vav*^ mice, we next sought to determine the effect of HIC1 deficiency in these specific cell populations during infection *C*. *rodentium*. We crossed *Hic1*^*fl/fl*^ mice with mice expressing Cre under the control of either the *Cd4* promoter or *Itgax* (CD11c) promoter to generate T cell-specific (*Hic1*^*CD4*^ mice) and dendritic cell-specific (*Hic1*^*CD11c*^ mice) HIC1-deficient mice. Both *Hic1*^*CD4*^ mice (Fig 3A–3C) and *Hic1*^*CD11c*^ mice (Fig 3D–3F) were as resistant to infection with *C*. *rodentium* as control *Hic1*^*fl/fl*^ mice, with equivalent weight loss, fecal bacterial burdens and expression of cytokines and antimicrobial peptide mRNA in the intestine. Thus, these results demonstrate that expression of HIC1 in T cells or CD11c-expressing cells is not required for immunity to bacterial infection and suggests loss of HIC1 in another cell population is responsible for the phenotype observed in *Hic1*^*Vav*^ mice.

### HIC1 expression in RORγt^+^ cells is critical for defence against intestinal bacterial infection

**Fig 4:**
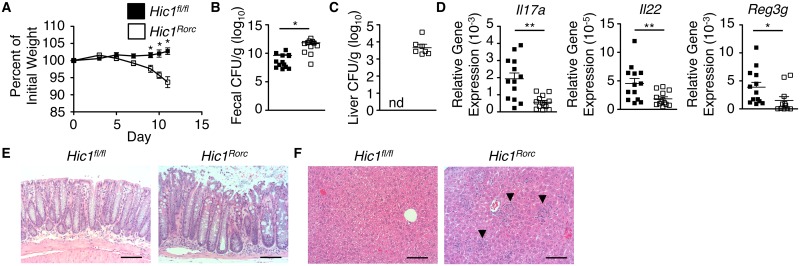
ILC3-intrinsic HIC1 is required for immunity to *Citrobacter rodentium* infection. *Hic1*^*Rorc*^ and *Hic1*^*fl/fl*^ mice were orally inoculated with *C*. *rodentium*. (A) Weight loss (percentage of initial weight) was calculated for each mouse over course of infection. (B, C) Bacterial loads (CFU/g) from fecal pellets (B) and liver (C) were measured at 11 days post inoculation (p.i.). (D) Quantitative RT-PCR was performed to determine expression of *Il17a*, *Il22* and *Reg3g* from distal colon tissue 11 days p.i. (E, F) H&E stained histological sections of colon (E) and liver (F) from 11 days p.i. Scale bar represents 100μm. Black arrows indicate inflammatory infiltrate. (A–D) Data are pooled from 3 independent experiments (*n* = 13–14 per group). *, P < 0.05; **, P < 0.01; Mann-Whitney test. Error bars indicate SEM. nd, none detected.

ILC3s have been shown to play a significant role in resistance to infection with *C*. *rodentium* [31,32]. To determine the role of HIC1 expression in RORγt^+^ ILC3s during infection with *C*. *rodentium*, we crossed *Hic1*^*fl/fl*^ mice with mice expressing Cre recombinase under the control of the *Rorc* promoter (*Hic1*^*Rorc*^ mice). Following infection with *C*. *rodentium*, and similar to what we observed in the *Hic1*^*Vav*^ mice, *Hic1*^*Rorc*^ mice displayed increased weight loss, higher fecal bacterial burdens and increased bacterial dissemination than control *Hic1*^*fl/fl*^ mice (Fig 4A–4C). Associated with increased susceptibility was reduced expression of *Il17a*, *Il22* and *Reg3g* in intestinal tissues (Fig 4D). We observed significant inflammation and tissue destruction in the intestine of infected *Hic1*^*Rorc*^ mice (Fig 4E), as well as inflammatory foci in the liver of *Hic1*^*Rorc*^ mice (Fig 4F). Thus, these results demonstrated that expression of HIC1 in RORγt^+^ cells is critical for immunity to *C*. *rodentium*.

**Fig 5:**
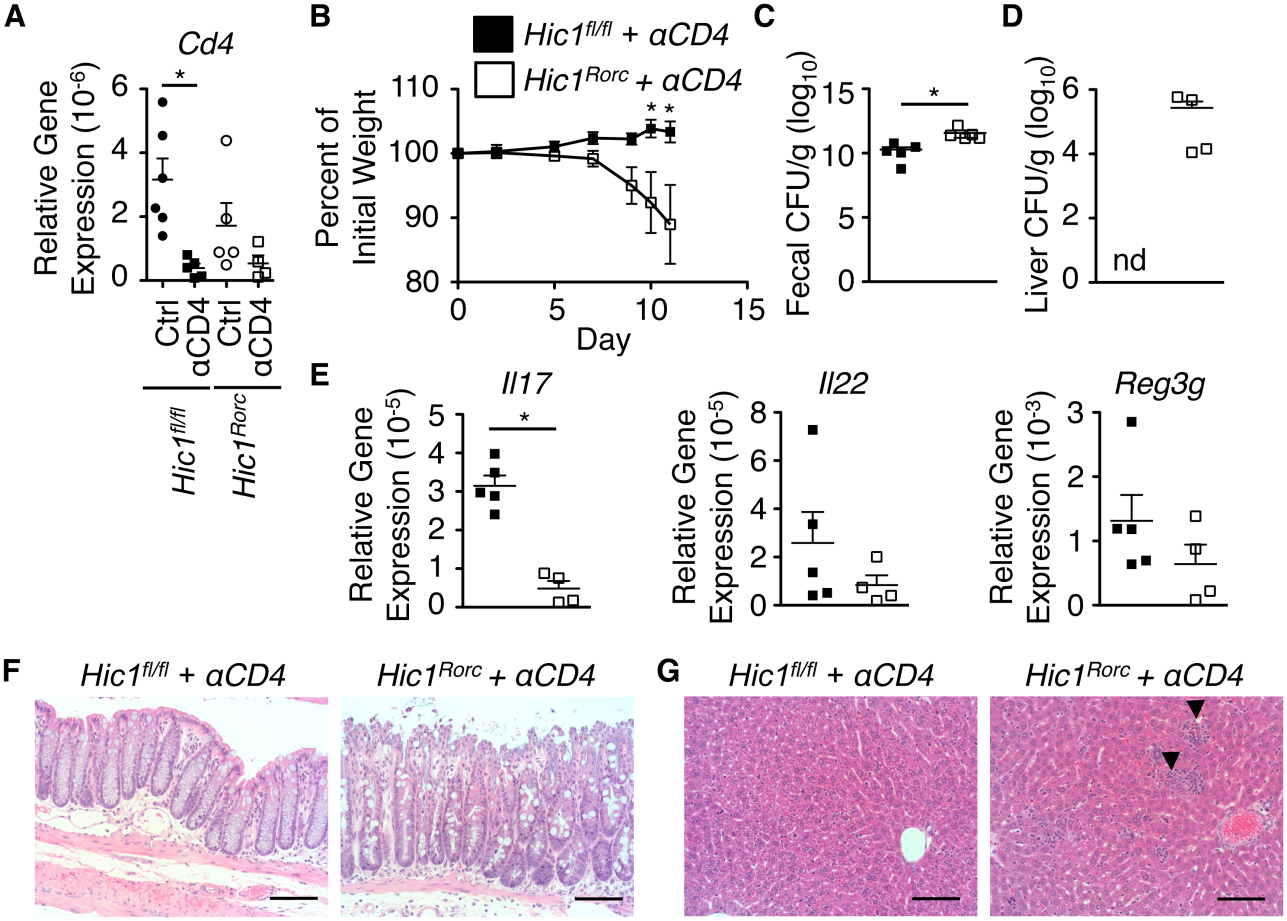
ILC3-intrinsic HIC1 is required for immunity to *Citrobacter rodentium* in T cell-depleted mice. *Hic1*^*Rorc*^ and *Hic1*^*fl/fl*^ mice were treated with a depleting anti-CD4 antibody and then orally inoculated with *C*. *rodentium*. (A) Colonic mRNA expression of *Cd4* in control and anti-CD4 antibody treated *Hic1*^*Rorc*^ and *Hic1*^*fl/fl*^ mice. (B) Weight loss (percentage of initial weight) was calculated for each mouse over course of infection. (C, D) Bacterial loads (CFU/g) from fecal pellets (C) and liver (D) were measured at 11 days post inoculation (p.i.). (E) Quantitative RT-PCR was performed to determine expression of *Il17a*, *Il22* and *Reg3g* from distal colon tissue 11 days p.i. (F, G) H&E stained histological sections of colon (F) and liver (G) from 11 days p.i. Scale bar represents 100μm. Black arrows indicate inflammatory infiltrate. Data from one experiment (***n*** = 4–6 per group). *, P < 0.05; Mann-Whitney test. Error bars indicate SEM. nd, none detected.

In addition to ILC3s, Cre expression in RORγt^+^ cells will drive deletion in T_H_17 cells. To remove any potential contribution of CD4^+^ T cells in the phenotype observed, we treated both *Hic1*^*fl/fl*^ and *Hic1*^*Rorc*^ mice with a depleting antibody against CD4 prior to infection with *C*. *rodentium* (Fig 5A). The absence of CD4^+^ cells had no significant effects on the differences observed during infection of *Hic1*^*fl/fl*^ and *Hic1*^*Rorc*^ mice, including weight loss (Fig 5B), fecal bacterial burden (Fig 5C), and bacterial dissemination and inflammation (Fig 5D–5G). Thus, the absence of HIC1 in RORγt^+^ ILC3s renders mice susceptible to *C*. *rodentium* infection.

### HIC1 is expressed by intestinal ILCs and is critical for intestinal immune homeostasis

**Fig 6:**
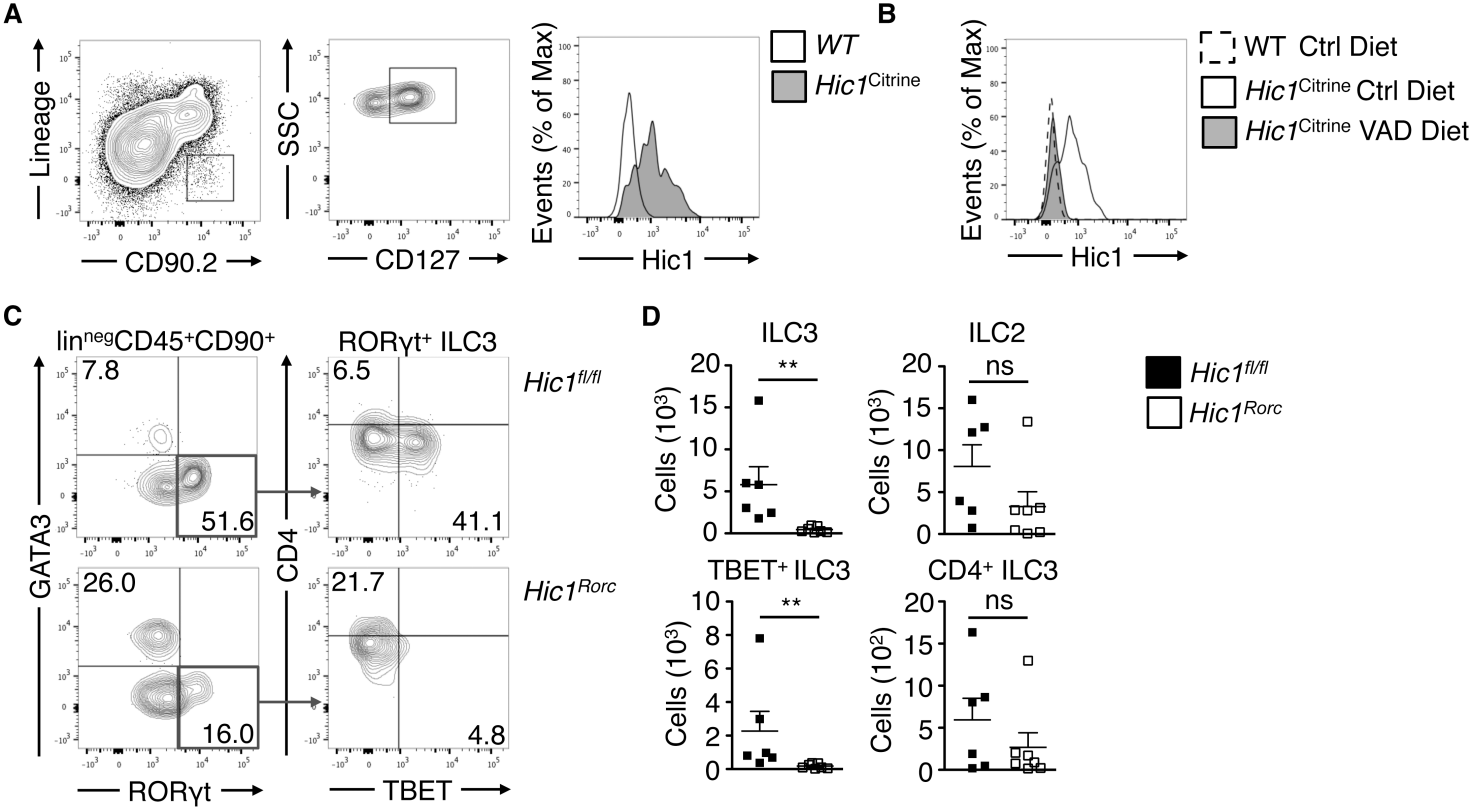
*Hic1* expression in intestinal ILCs is vitamin A dependent and is required for intestinal immune homeostasis. (A) ILCs (lin^neg^ CD90^+^ CD127^+^ cells) were analyzed by flow cytometry for *Hic1*^*Citrine*^ reporter expression from the intestinal lamina propria (LP). Data representative of 2 independent experiments (B) *Hic1* reporter expression in intestinal LP ILCs (lin^neg^ CD90^+^ CD127^+^ cells) from *Hic1*^*Citrine*^ mice fed a control diet, *Hic1*^Citrine^ mice fed a vitamin A deficient (VAD) diet, and controls fed a control diet was analyzed by flow cytometry. Data are representative of 2 independent experiments (*n* = 4–5 per group). (C, D) Intestinal LP cells from *Hic1*^*fl/fl*^ and *Hic1*^*Rorc*^ mice at steady state were analyzed by flow cytometry to enumerate populations of ILC3s (RORγt^+^) and ILC2s (GATA3^+^). Data are from 3 independent experiments (*n* = 6–7 per group) *, P < 0.05; **, P < 0.01; Mann-Whitney test. Error bars indicate SEM.

Our results suggest that HIC1 expression in ILC3s is critically important for immunity to intestinal bacterial infection. Using mice with a fluorescent reporter gene inserted in the *Hic1* locus (*Hic1*^Citrine^ mice) [33] we determined that in addition to previously identified populations including T cells, dendritic cells and macrophages [26], lineage-negative (lin^neg^) CD90.2^+^ CD127^+^ ILCs isolated from the intestinal LP express HIC1 (Fig 6A), which was dependent on the availability of atRA, as *Hic1*^Citrine^ mice reared on a VAD diet did not express HIC1 in ILCs within the LP (Fig 6B). Loss of HIC1 in RORγt^+^ cells (in *Hic1*^*Rorc*^ mice) resulted in a specific change in ILC populations in the LP. In the steady state, we observed significantly fewer RORγt^+^ ILCs (ILC3s) in the LP of *Hic1*^*Rorc*^ mice, with a significant reduction in the number of RORγt^+^ TBET^+^ ILC3s (Fig 6C and 6D). We found no change in the number of CD4^+^ ILC3s (also known as lymphoid tissue inducer (LTi) cells) nor in numbers of the canonical GATA3^+^ ILC (ILC2) population (Fig 6C and 6D).

### HIC1 does not regulate ILC precursors in the bone marrow

**Fig 7:**
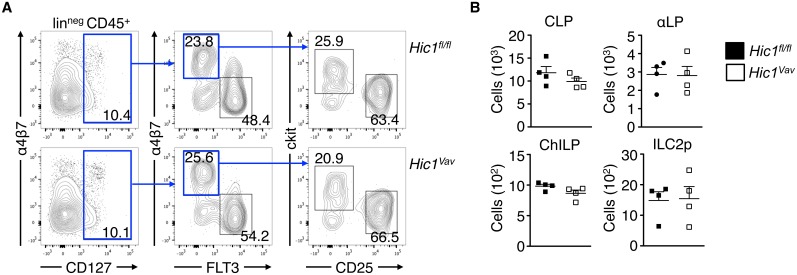
*Hic1* does not regulate ILC precursors in the bone marrow. (A) Gating strategy and (B) cell numbers of CLPs (CD45^+^ lin^neg^ CD127^+^ Flt3^+^ α4β7^−^), α4β7^+^ lymphoid progenitors (αLP; CD45^+^ lin^neg^ CD127^+^ Flt3^−^ α4β7^+^), ChILPs (CD45^+^ lin^neg^ CD127^+^ Flt3^−^ α4β7^+^ CD25^−^ c-Kit^+^) and ILC2 progenitors (ILC2p; (CD45^+^ lin^neg^ CD127^+^ Flt3^−^ α4β7^+^ CD25^+^ c-Kit^−^) from bone marrow of *Hic1*^*Vav*^ and *Hic1*^*fl/fl*^ mice. Data are from two independent experiments (*n* = 4 per group). *, P < 0.05; Mann-Whitney test. Error bars indicate SEM.

As we observed a significant reduction of ILC3s in the LP in the absence of HIC1, we next tested whether the lack of HIC1 affected the upstream development of ILC precursors in the bone marrow. ILCs develop in the bone marrow through a lineage pathway that begins with a common lymphoid progenitor (CLP) and progresses through an α4β7-expressing lymphoid progenitor (αLP), a common progenitor to all helper-like ILCs (ChILP) and, in the case of ILC2s, an ILC2 precursor (ILC2p) [34]. Analysis of surface marker expression on lineage-negative, CD45^+^ bone marrow cells showed that HIC1 was not required for the development of CLP, αLP, ChILP, or ILC2p populations (Fig 7A and 7B). Thus, the reduced number of ILC3s in the LP is not due to a reduced frequency of ILC precursors and suggests that HIC1 is required for ILC3 homeostasis in the periphery.

### IL-22 treatment of *Hic1*^*Rorc*^ mice promotes immunity to *C*. *rodentium*

**Fig 8:**
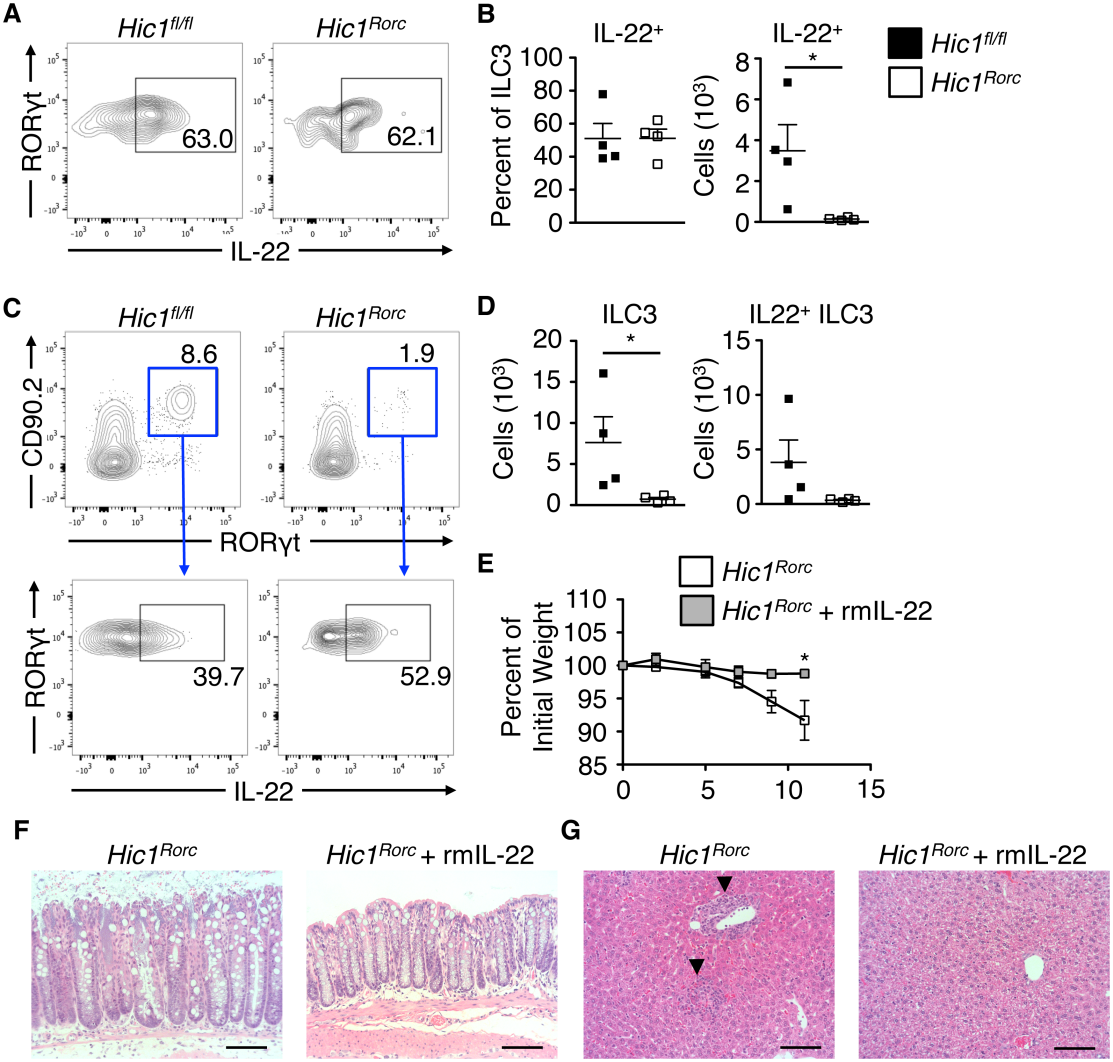
Recombinant mouse IL-22 is sufficient to promote resistance to *Citrobacter rodentium* in *Hic1*^*Rorc*^ mice. (A-D) Intestinal ILC3s from *Hic1*^*Rorc*^ and *Hic1*^*fl/fl*^ mice were analysed for intracellular IL-22 by flow cytometry at steady state (A, B) or at day 4 post infection with *C*. *rodentium* (C, D). (E–G) *Hic1*^*Rorc*^ mice were infected with *C*. *rodentium* and treated with or without rmIL-22. (E) Weight loss (percentage of initial weight) was calculated for each mouse over course of infection. H&E stained histological sections of colon (F) and liver (G) from 11 days post infection. Scale bar represents 100μm. Black arrows indicate inflammatory infiltrate. (A–D) Data are from 2 independent experiments (*n* = 4 per group) (E) Data are from 2 independent experiments (*n* = 5–6 mice per group). *, P < 0.05; Mann-Whitney test. Error bars indicate SEM.

IL-22 production by innate immune cells is critically important for immunity to *C*. *rodentium* [35]. We observed a significant reduction in IL-22-producing ILC3s in naïve *Hic1*^*Rorc*^ mice (Fig 8A and 8B) and infection with *C*. *rodentium* failed to expand the small number of ILC3s in *Hic1*^*Rorc*^ mice (Fig 8C and 8D). We hypothesized that the reduced levels of IL-22 were responsible for susceptibility to infection. Treatment of *Hic1*^*Rorc*^ mice with recombinant IL-22 on days -2, -1, 0, 1, 3, 5 and 7 during *C*. *rodentium* infection resulted in significant protection from infection, as measured by reduced weight loss (Fig 8E), less intestinal pathology (Fig 8F) and a lack of bacterial dissemination to the liver (Fig 8G). Thus, the RA–HIC1 axis is critical for immunity to intestinal bacterial infection by regulating IL-22-producing ILC3s in the intestine.

## Discussion

Our results demonstrate that in the steady state, HIC1 is expressed by intestinal ILCs in a Vitamin A-dependent manner. In the absence of HIC1, we observed a dramatic decrease in intestinal ILC3 numbers, which was associated with a failure to clear *C*. *rodentium* infection. Together, these results highlight an important role for HIC1 not only in regulating intestinal immune homeostasis but also in mounting proper immune responses to an intestinal bacterial infection.

In the absence of HIC1, we found a significant reduction in the number of ILC3s with no effect on ILC2s in the intestine. Specifically, there were reduced numbers of RORγt^+^ TBET^+^ ILC3s that produce IL-22 with no difference in CD4^+^ ILC3s (LTi). This is consistent with studies that have demonstrated that these two lineages have distinct developmental pathways; LTi cells develop in the fetus while TBET^+^ ILC3s develop postnatally and rely on environmental signals [10,36,37]. Interestingly, it has been shown that atRA signalling is also important for generation of LTi cells in the fetus [38]. However, our results suggest that HIC1 is not involved in fetal LTi formation, as we find no differences in LTi numbers or lymphoid structures in the absence of HIC1. Further, the development of ILC progenitor cells in the bone marrow is not perturbed by loss of HIC1, suggesting that the primary role of HIC1 is to regulate the development and function of adult cells in the periphery.

Although we have yet to define the precise molecular mechanisms of HIC1-dependent regulation of intestinal ILC3 function, the RA-HIC1 axis may be acting to control cellular quiescence. atRA has been shown to control hematopoietic stem cell dormancy, a profound state of quiescence, that is counteracted by activation of Myc-dependent proliferation [39,40]. As we have previously observed in intestinal T cells, HIC1 is dispensable for the expression of intestinal homing markers and migration to the intestine [26]. Thus, our current working hypothesis is that RA induces expression of HIC1 to promote cellular quiescence/dormancy in the intestinal microenvironment, possibly by regulating Myc-dependent processes including metabolism and proliferation. Future studies will examine if HIC1 has a similar role in ILC3s.

Resistance to intestinal infection with *C*. *rodentium* is mediated by IL-22, and ILC3s are the predominant IL-22-producing cell population during the first week of infection [32,41]. There are contradictory studies on which ILC3 populations are key for resistance to *C*. *rodentium* with both CD4^+^ LTis and natural cytotoxicity receptor (NCR)^+^ ILC3s each being described as either individually critical or redundant [32,42,43]. Another study looking at TBET^+^ ILC3s (which include NCR^+^ ILC3s) demonstrated that TBET expression in a subset of ILC3s is critical for resistance to *C*. *rodentium* infection [44]. Our results are consistent with a role for NCR^+^ or TBET^+^ ILC3s in immunity to *C*. *rodentium* as *Hic1*^CD4^ mice (deficient for HIC1 in T cells and LTi) are resistant to infection while *Hic1*^*Rorc*^ mice (deficient for HIC1 in T_H_17 cells and all ILC3s) are susceptible. Further, depletion of CD4-expressing cells in *Hic1*^*Rorc*^ mice had no effect on disease development. Thus, HIC1 expression in ILC3s is critical for immunity to *C*. *rodentium*.

Taken together, these results establish a role for the transcriptional repressor HIC1 as an atRA-responsive cell-intrinsic regulator of ILC3 cell function in the intestine, and identify a potential regulatory pathway that could be targeted to modulate ILC3 responses in the intestine.

## Methods

### Ethics statement

Experiments were approved by the University of British Columbia Animal Care Committee (Protocol A13-0010) and were in accordance with the Canadian Guidelines for Animal Research.

### Mice

The generation of *Hic1*^Citrine^ mice has been described [33] and *Hic1*^*fl/fl*^ mice will be described elsewhere (manuscript in preparation). *Cd4*-Cre mice were obtained from Taconic, *Vav*-Cre mice were obtained from T. Graf (Centre for Genomic Regulation, Barcelona, Spain) and *CD11c*-Cre (B6.Cg-Tg(Itgax-cre)1-1Reiz/J) and RORc-Cre (B6.FVB-Tg(RORc-cre)1Litt/J) mice were obtained from the Jackson Laboratory (Bar Harbor, ME, USA). Animals were maintained in a specific pathogen-free environment at the UBC Biomedical Research Centre animal facility.

### Diet studies

Vitamin A-deficient (TD.09838) diet was purchased from Harlan Teklad Diets. At day 14.5 of gestation, pregnant females were administered the vitamin A-deficient diet and maintained on diet until weaning of litter. Upon weaning, females were returned to standard chow, whereas weanlings were maintained on special diet until use.

### Isolation of lamina propria lymphocytes

Peyer’s patches were removed from the small intestine, which was cut open longitudinally, briefly washed with ice-cold PBS and cut into 1.5 cm pieces. Epithelium was stripped by incubation in 2mM EDTA PBS for 15 minutes at 37°C and extensively vortexed. Remaining tissue was digested with Collagenase/Dispase (Roche) (0.5 mg/mL) on a shaker at 250 rpm, 37°C, for 60 minutes, extensively vortexed and filtered through a 70μm cell strainer. The flow-through cell suspension was centrifuged at 1500rpm for 5 min. The cell pellet was resuspended in 30% Percoll solution and centrifuged for 10 minutes at 1200 rpm. The pellet was collected and used as lamina propria lymphocytes.

### Antibodies and flow cytometry

Absolute numbers of cells were determined via hemocytometer or with latex beads for LP samples. Intracellular cytokine (IC) staining was performed by stimulating cells with 50 ng/ml phorbol 12-myristate 13-acetate (PMA), 750 μg/ml ionomycin, and 10 μg/ml Brefeldin-A (Sigma, St. Louis, MO) for 4 hours and fixing/permeabilizing cells using the eBioscience IC buffer kit. All antibody dilutions and cell staining were done with phosphate-buffered saline (PBS) containing 2% fetal calf serum (FCS), 1 mM Ethylenediaminetetraacetic acid (EDTA), and 0.05% sodium azide. Fixable Viability Dye eFluor 506 was purchased from eBioscience (San Diego, CA) to exclude dead cells from analyses. Prior to staining, samples were Fc-blocked with buffer containing anti-CD16/32 (93, eBioscience) and 1% rat serum to prevent non-specific antibody binding. Cells were stained with fluorescent conjugated anti-CD11b (M1/70), anti-CD11c (N418), anti-CD19 (ID3), anti-CD5 (53–7.3), anti-CD8 (53.67), anti-CD3 (KT3)(2C11), anti-NK1.1 (PK136), anti-B220 (atRA-6B2), anti-Ter119 (Ter119), anti-Gr1 (RB6-8C5) produced in house, anti-CD4 (GK1.5), anti-CD45 (30-F11), anti-CD90.2 (53–2.1), anti-GATA3 (TWAJ), anti-RORγt (B2D), anti-TBET (eBio4B10), anti-FLT3 (A2F10), anti-CKIT (ACK2), anti-TCRβ (H57-597), anti-TCRγδ (eBioGL3), anti-MHCII (I-A/I-E) (M5/114.15.2), anti-F4/80 (BM8), anti-α4β7 (DATK32), anti-IL-22 (IL22JOP), anti-Ki67 (SolA15), anti-CCR9 (eBioCW-1.2) purchased from eBioscience, anti-CD127 (5B/199), anti-CD64 (X54.5/7.1.1) purchased from BD Biosciences. Data were acquired on an LSR II flow cytometer (BD Biosciences) and analysed with FlowJo software (TreeStar).

### *Citrobacter rodentium* infection

Mice were infected by oral gavage with 0.1 ml of an overnight culture of Luria-Bertani (LB) broth grown at 37°C with shaking (200 rpm) containing 2.5 x 10^8^ cfu of C. rodentium (strain DBS100) (provided by B. Vallance, University of British Columbia, Vancouver, British Columbia, Canada). Mice were monitored and weighed daily throughout the experiment and sacrificed at various time points. For enumeration of *C*. *rodentium*, fecal pellets or livers were collected in pre-weighed 2.0 ml microtubes containing 1.0 ml of PBS and a 5.0 mm steel bead (Qiagen). Tubes containing pellets or livers were weighed, and then homogenized in a TissueLyser (Retche) for a total of 6 mins at 20 Hz at room temperature. Homogenates were serially diluted in PBS and plated onto LB agar plates containing 100 mg/ml streptomycin, incubated overnight at 37°C, and bacterial colonies were enumerated the following day, normalizing them to the tissue or fecal pellet weight (per gram). Colon tissues were fixed overnight in 10% buffered formalin and paraffin-embedded. A total of 5-μm-thick tissue sections were stained with hematoxylin and eosin (H&E) for histological analysis. In some cases, mice were treated with 400 ng recombinant mouse IL-22 (Biolegend) by i.p. injection daily for 4 days starting 2 days prior to infection. Injections with rmIL-22 continued every other day following day 1 post infection. In other cases, mice were injected i.p. on days -1, 2, 5 and 8 post infection with 500 μg of anti-CD4 (GK1.5) (produced in-house by AbLabBiologics, UBC (Vancouver, BC)), constituted in sterile PBS.

### RNA isolation and quantitative real-time PCR

Tissues were mechanically homogenized and RNA was extracted using the TRIzol method according to the manufacturer's instructions (Ambion). cDNA was generated using High Capacity cDNA reverse transcription kits (Applied Biosystems). Quantitative PCR was performed using SYBR FAST (Kapa Biosystems) and SYBR green-optimized primer sets run on an ABI 7900 real-time PCR machine (Applied Biosystems). Cycle threshold (C_T_) values were normalized relative to beta-actin (*Actb*) gene expression. The primers used were synthesized de novo:

Il17a
forward 5’-AGCAGCGATCATCCCTCAAAG-3’
reverse 5’-TCACAGAGGGATATCTATCAGGGTC-3’;
Il22
forward 5’-ATGAGTTTTTCCCTTATGGGGAC-3’
reverse 5’-GCTGGAAGTTGGACACCTCAA-3’
Reg3g
forward 5’-CCGTGCCTATGGCTCCTATTG-3’
reverse 5’-GCACAGACACAAGATGTCCTG-3’
Actb
forward 5’-GGCTGTATTCCCCTCCATCG-3’
reverse 5’-CCAGTTGGTAACAATGCCATGT-3’

### Statistics

Data are presented as mean ± S.E.M. A two-tailed Mann-Whitney test using GraphPad Prism 5 software determined statistical significance. Results were considered statistically significant with *P* < 0.05.
